# Mesenchymal Stem Cells Promoted Lung Wound Repair through Hox A9 during Endotoxemia-Induced Acute Lung Injury

**DOI:** 10.1155/2017/3648020

**Published:** 2017-03-29

**Authors:** Xi Xin, Liu Yan, Zhu Guangfa, Huang Yan, Li Keng, Wu Chunting

**Affiliations:** ^1^Department of Pulmonary and Critical Care Medicine, Beijing Anzhen Hospital, Capital Medical University, Beijing Institute of Heart, Lung and Blood Vessel Diseases, Beijing 100029, China; ^2^Department of Infectious Diseases, Beijing Anzhen Hospital, Capital Medical University, Beijing Institute of Heart, Lung and Blood Vessel Diseases, Beijing 100029, China

## Abstract

*Objectives*. Acute lung injury (ALI) is a common clinical critical disease. Stem cells transplantation is recognized as an effective way to repair injured lung tissues. The present study was designed to evaluate the effects of mesenchymal stem cells (MSCs) on repair of lung and its mechanism.* Methods*. MSCs carrying GFP were administrated via trachea into wild-type SD rats 4 hours later after LPS administration. The lung histological pathology and the distribution of MSCs were determined by HE staining and fluorescence microscopy, respectively. Next, differentially expressed HOX genes were screened by using real-time PCR array and abnormal expression and function of Hox A9 were analyzed in the lung and the cells.* Results*. MSCs promoted survival rate of ALI animals. The expression levels of multiple HOX genes had obvious changes after MSCs administration and HOX A9 gene increased by 5.94-fold after MSCs administration into ALI animals. HOX A9 was distributed in endothelial cells and epithelial cells in animal models and overexpression of Hox A9 can promote proliferation and inhibit inflammatory adhesion of MSCs.* Conclusion*. HoxA9 overexpression induced by MSCs may be closely linked with lung repair after endotoxin shock.

## 1. Introduction

Acute lung injury (ALI) is a common critical disease in intensive care units (ICU) and a big problem in the modern critically ill medicine. It is usually characterized by the injury of lung epithelial and endothelial barrier, diffuse lesions of pulmonary capillaries with enhanced permeability, neutrophils into lung tissues, and imbalances of the lungs proinflammatory and anti-inflammatory factor [1, 2]. ALI is easy to develop into severe acute respiratory distress syndrome (ARDS), with progressive respiratory distress and refractory hypoxemia as clinical features [3]. Current clinical treatments include incentives removal, infection control, lung-protective ventilation as soon as possible, and organ supportive therapy to maximize the reduction of the number and degree of cellular damage; however, there is no real breakthrough on the reduction of incidence and overall mortality of ARDS [4]. Therefore, looking for a new and effective method for the treatment of ALI has become an important subject to be studies.

In recent years, stem cell transplantation has always been the hot spot of the international research. At present, there are two broad categories in the field of stem cell transplantation, commonly used embryonic stem cells and adult stem cells. Embryonic stem cells is difficult to be used in clinic for its ethics and tumorigenic action. However, the adult stem cells have the characteristics of multiple cellular differentiation potential without ethic problem. Studies have shown that stem cells can be used as a biological marker of prognosis and a new treatment method, because they have the advantage of targeting the injured tissues, differentiating into the right cells, and repairing damage area [5]. In addition, more and more reports think that stem cells may secrete various cytokines and growth factors, which play an important role in wound repair. Mesenchymal stem cells (MSC) is a kind of adult stem cells and the most studied stem cells in lung injury repair, which can differentiate into alveolar, bronchial epithelial, and endothelial cells [6, 7]. Lung endothelium and epithelium have been considered as the first and key barrier against injuries [8].

- Homebox (HOX) gene is a highly conservative evolutionary transcription factors and all their coding products contain a DNA-binding domain composed of about 60 amino acids, which target DNA sequence and modulate gene expression [9]. Reports showed that HOX gene plays a very important role not only in embryonic development but in vascular repair, angiogenesis, and tumor metastasis after birth [10, 11]. Research has shown that HOX genes participates in a variety of cell proliferation and migration by direct or indirect way in lung development [8, 9]. Moreover, Hox genes are closely related with the differentiation of stem cells such as Hoxa3 and Hoxd3 drive angiogenesis and endothelial cell sprouting in adults and promote embryonic stem cells differentiation towards endothelial cells [10]. However, the differential expression and precise role of HOX expression in ALI after MSCs administration remains unknown.
- Considering the important role of HOX in differentiation of stem cells, in the present study, we aimed to display protective effects of MSCs on LPS-induced ALI animal models and the Hox-expressing character during protective process. The effects of upregulated HOXA9 after MSCs administration to ALI animals were also examined.

## 2. Materials and Methods

### 2.1. Ethics Statement

Wild-type SD rats (6–8 weeks) were supplied by Laboratory Animal Center, Beijing, China, and maintained under specific pathogen-free condition (Animal Center, Capital Medical University, Beijing, China). All experiments referring to the use of animals were approved by the Committee of Animal Care and Use of Capital Medical University.

### 2.2. Rat Mesenchymal Stem Cells and Cell Culture

Rat MSCs were purchased from Cyagen Bioscience, Inc. (Guangzhou, China), which were derived from normal rat bone marrow. They expressed CD90, CD44, and CD29, but not CD34, CD45, and CD11b/c through flow cytometry assay, which was supplied by suppliers. The MSC cells were grown in SD rat MSC basal medium (Cyagen Bioscience, Inc., Guangzhou, China) supplemented with 10% SD rat MSC-qualified fetal bovine serum, 1% penicillin-streptomycin, and 1% glutamine at 37°C in 5% CO_2_ and saturated humidity. The growth medium was changed every two days. When the cells are approximately 80–90% confluent, they can be dissociated with Trypsin-EDTA and passaged. Lentivirus carrying GFP (pLenO-GFP) was offered by GenePharma (Shanghai, China). MSCs were infected by pLenO-GFP with a MOI = 50 and cultured in a 6-well plate (10^6^ cells/well) with 5% CO_2_ at 37°C. The culture medium was changed 24 hours later. The infection efficiency of pLenO-GFP was identified by fluorescence microscopy at 48 hours after transfection (Olympus Co., Tokyo, Japan).

### 2.3. LPS-Induced ALI in Rats

Wild-type SD rats (weighing 200 to 250 g) were challenged by LPS (10 mg/kg,* Escherichia coli* 0111:B4, Sigma, MO, USA) dissolved in 100 *μ*L of PBS to induce ALI via intraperitoneal injection, followed by PBS (100 *μ*L) or MSC (2 × 10^6^ cells resuspended in 100 *μ*L PBS) infusion via intratracheal instillation 4 hours later after LPS challenge. Grouping was as follows: normal group, normal + MSC group, LPS group, and LPS + MSC group. The animals were given analgesic (Buprenorphine, i.p. 0.1 mg/kg, ACRIS, Germany) twice per day to minimize sufferings. Rats were sacrificed at 24 hours after PBS or MSC infusion. Lung lobes were obtained for further analysis.

### 2.4. Lung Histological Pathology

To evaluate the severity of lung injury, the right lobe lungs were obtained at 24 hours after MSCs infusion. The lung tissues were then fixed in 4% paraformaldehyde for 24 hours. After fixation, lung tissues were embedded in paraffin and cut into 5 *μ*m sections. Then the sections were examined by HE staining. At the same time, the distribution of MSCs in the lungs was observed using fluorescence microscopy at 24 hours (Olympus Co., Tokyo, Japan).

### 2.5. Homeobox (HOX) Genes PCR Array

Rat Homeobox (HOX) Genes PCR Array was supplied by QIAGEN, USA. RNA was isolated by using TRIZOL® Reagent (Invitrogen, USA) and then incubated in DNase I solution at 37°C for 30 minutes to remove contaminated DNA. Next, the RNA was cleaned up using RNeasy® MinElute™ Cleanup Kit (Qiagen, USA). RNA yield and quality were assayed by detecting the ratio of A260 to A280 values and denaturing agarose gel electrophoresis. After first Strand cDNA Synthesis by using SuperScript III Reverse Transcriptase (invitrogen, USA), real Real-Time PCR was performed by using 2x SuperArray PCR master mix in 96-Well PCR Arrays at 95°C, 10 min, then 95°C, 15 s, 60°C, 1 min for 40 amplification cycles. The fold-change was calculated for each gene from experimental to control as 2^−ΔΔCt^.

### 2.6. Immunohistochemistry

The slides were stained with rabbit anti-rat HoxA9 primary antibody (1 : 200, Santa Cruz, CA, USA) at room temperature for 1 h after hydrogen peroxide treatment for 20 minutes to remove the activity of endogenous peroxidase. Then, the sections were washed in PBS and then incubated with biotinylated goat anti-rabbit IgG for 1 h and washed again. After washing in PBS, the signal was detected with 3,3′-diaminobenzidine (Dingguo, Beijing, China).

### 2.7. Construction of pLenO-HoxA9

The full coding sequence of HoxA9 was cloned into EcoR I and BamH I of plasmid pLenO-GFP (GenePharma, Shanghai, China). The constructed plasmids were verified by restriction enzyme mapping and DNA sequencing. Production of pLenO-HoxA9 was performed using the combined ratio of transfer plasmid, packaging plasmid, Env plasmid, and pRSV-Rev plasmid at 4 : 2 : 2 : 1 by using CaCl_2_. A collection was made after 48 hrs and was cleared by centrifugation at 1500 rpm for 5 min at 4°C. Concentration of lentivirus using ultracentrifugation was performed for 90 min at 100,000 rpm. Supernatant was completely removed and virus pellets were resuspended in PBS overnight at 4°C and stored at −80°C until use. Titers were determined by fluorescence microscopy after transducing pLenO-HoxA9 into 293 T cells with different concentrations [11].

### 2.8. Quantitative Real-Time PCR

MSCs treated by pLenO-GFP or pLenO-HoxA9 were obtained and cultured in cell culture medium, respectively. Total RNA was isolated using TRIzol reagent (Introgen, USA) according to the manufacturer's instruction. The RT-PCR assays were performed following the instruction for Power qPCR PreMix (SYBR Green) described by Generay Biotech Inc. (Shanghai, China). Primers used for the real-time PCR were supplied by Dingguo, Beijing. The sequences of the PCR primers used were the forward and reverse primer sequences for GADPH which were 5′-CCGCAGCCTCGTCTCATAGAC-3′ and 5′-TTGACTGTGCCGTTGAACTTGC-3′. The forward and reverse primer sequences for HoxA9 were 5′-CTTTGTCCCTGACTGACTATGCTTG-3′ and 5′-TCTTCTAGTTGTTCCTGGCTCGT-3′.

### 2.9. Proliferation Assay

MSCs from control group, empty vector group, or Hox A9 expressed group were harvested and washed twice in PBS, then fixed in 1 mL cold 70% ethanol, and fixed at 4°C overnight. The fixed cells were washed twice with PBS, stained in a propidium iodide solution with a final concentration (50 ug/mL, Sigma USA) for 1 hour, and treated with a ribonuclease A solution (20 ug/mL, sigma USA) at 37°C for 30 minutes. Flow cytometry (BD Pharmingen, USA) was then used to examine cell cycle.

### 2.10. Leukocyte-MSCs Adherence Assay

Glucan400 (4%) saline solution was added to the rat whole blood with a dilution of 2 : 1 at room temperature for 1 h. The supernatant rich in leukocytes was obtained after centrifugation at 1500 rpm for 10 min and layered on lymphocyte separation medium with a volume ratio of 1 : 1 (density 1.077 g/mL, Dingguo, China). Following subsequent centrifugation at 2000 r/min for 10 min, the leukocyte layer at the interface was collected and resuspended in complete medium at a final density of 1 × 10^7^ cells/mL. The cell viability (>99%) was determined by trypan blue staining. Then, the medium of MSCs in 6-well plates (10^6^ cells/well) was removed and the leukocytes (2 × 10^5^ cells/well) were then added to a total volume of 2.0 mL MSC medium. After 24 h of incubation at 37°C, nonadherent cells were removed by washing twice with PBS and the remaining cells were collected and incubated with PE anti-rat CD45 antibody (BD Biosciences, CA, USA) to stain the leukocytes for 20 min at 25°C. After washing, the cells were resuspended in 0.5 mL of PBS and analyzed on a flow cytometer (BD Pharmingen, USA).

### 2.11. Statistical Analysis

Data are shown as the means ± standard deviations (SD). The intergroup comparison used ANOVA and paired* t*-test, and the pairwise comparison among multiple groups used the LSD test. Statistical analyses were performed using SPSS 16.0 software. A value of *P* < 0.05 was considered to be statistically significant.

## 3. Results

### 3.1. MSCs Ameliorate ALI in a LPS-Induced Animal Model

**Figure 1:**
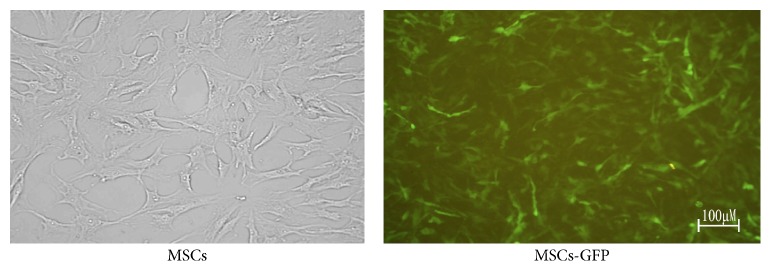
MSCs carrying GFP were determined by fluorescence microscopy. The transfection efficiency was over 80%.

Proliferation of MSCs accelerated significantly after 6 generations with cells arranged in order. Lentivirus transfection (MOI = 50) did not affect the proliferation and morphology of MSCs. The GFP positive cells were above 80% by counting under fluorescence microscopy (Figure 1).

**Figure 2:**
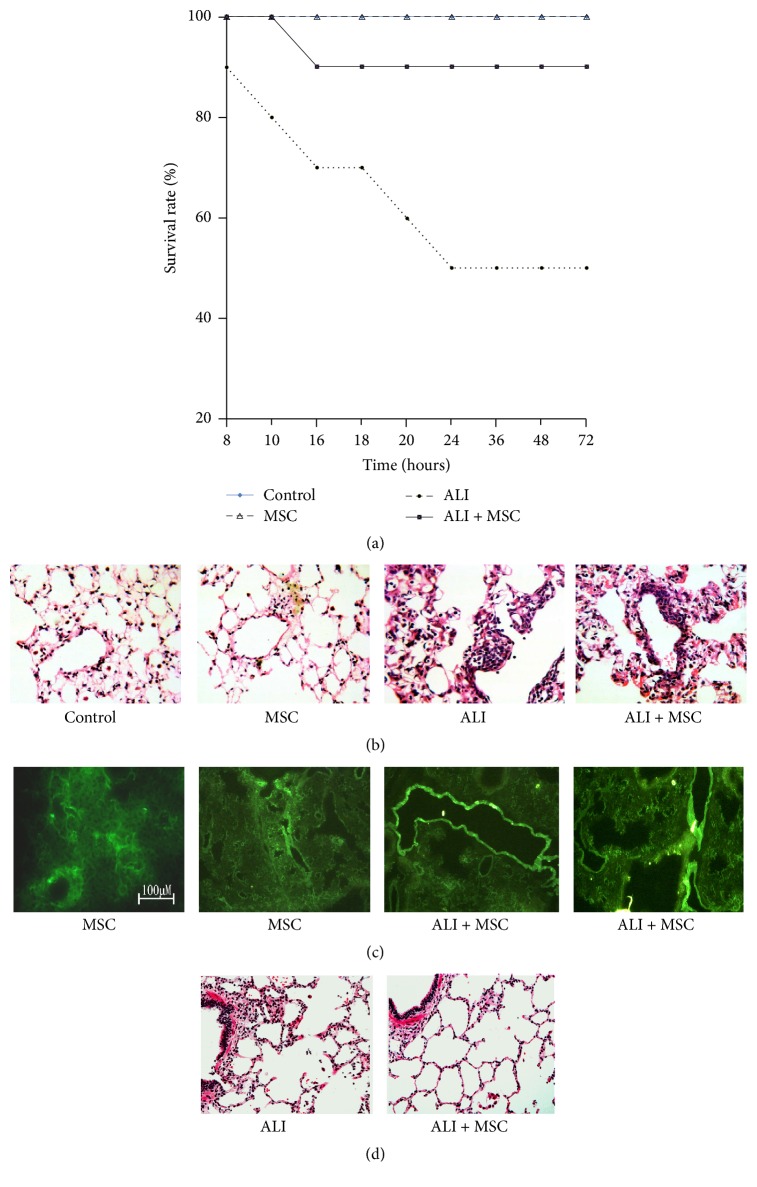
Protective action of MSCs was determined by survival rate and HE staining. (a) The survival assay. The survival rate was nearly 50% at 72 h, and administration of MSCs significantly increased survival rate to 90%. (b) HE staining at 24 hours after MSCs administration. The lung tissues from the LPS group demonstrated significantly pathological alterations, which was attenuated by MSCs administration. (c) Distribution of MSCs at 24 hours after MSCs administration. Fluorescence was distributed throughout the distal lungs when administrating MSCs into normal rats; however, the fluorescence was mainly distributed in larger blood vessels when administrating MSCs into ALI Rats. (d) HE staining at 7 days after MSCs administration. Lung injury was obviously attenuated by MSCs administration.

Then, MSCs carrying GFP were administrated via tracheal instillation into LPS-induced ALI animal model. All rats in the normal group survived. In the LPS-challenge group, the survival rate was nearly 50% at 72 h, and administration of MSCs significantly increased the survival rate to 90% (Figure 2(a)). To observe the pathological changes of the lung tissues, HE staining was utilized in our study. As shown in Figure 2(b), at 24 hours after MSCs administration, the lung tissues from the LPS group demonstrated significantly pathological alterations, including notable inflammatory cells infiltration, interstitial and intra-alveolar edema, and some collapsed alveoli which was attenuated by MSCs administration. Fluorescence at 24 hours after MSCs administration was measured in lungs after MSCs administration using fluorescence microscopy. As shown in Figure 2(c), fluorescence was distributed throughout the distal lungs when administrating MSCs into normal rats; however, the fluorescence was mainly distributed in larger blood vessels when administrating MSCs into ALI rats. At 7 days after MSCs administration, lung injury was obviously attenuated by MSCs administration.

### 3.2. HOX Gene Expression in ALI after MSC Administration

Expression of HOX was detected by real-time PCR array after 24 h of MSC administration. More than 2-fold change was recognized as differentially expressed genes. When compared with normal group, there were 6 upregulated HOX genes in MSC group (Hhex, Hox a5, Hoxb5, Hoxb7, Hox c9, and Lhx2). There were 3 upregulated HOX genes (Hoxa1, Hox b5, and Meis1) and 1 downregulated gene (Hoxa5) in ALI group. There were 8 differential expression of HOX genes with 6 upregulated (Hhex, Hoxa1, Hoxa9, Hoxb7, Hox c9, and Lhx2) and 2 downregulated (HOX a3 and Hoxd8) in MSC + ALI group. When compared with ALI group, there were 8 differential expressed HOX genes including Hhex, Hoxa3, Hoxa5, Hoxa9, Hoxb5, Hox d8, Lhx2, and Meis1. Among them, HoxA9 increased at 24 after MSC administration with more than tenfold (Table 1).

### 3.3. Detection of HoxA9 Expression in the ALI Lung after MSC Administration

**Figure 3:**
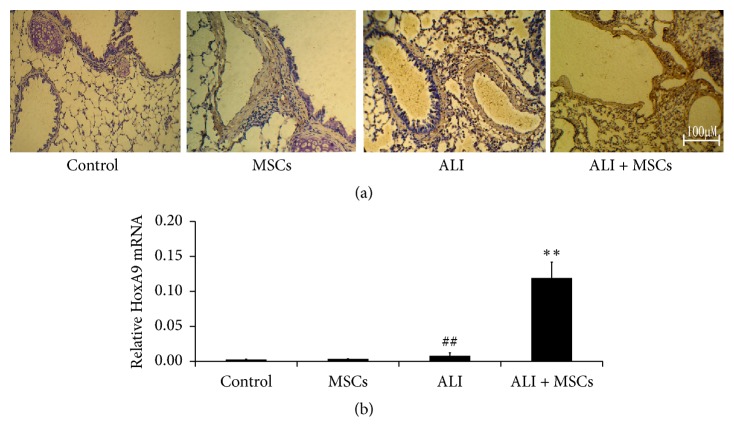
HoxA9 expression in lung was determined by immunohistochemistry (a) and real-time PCR (*n* = 4, (b)). HoxA9 expression in the ALI + MSCs group was increased markedly at 24 hours and the increase of HoxA9 expression was significantly distributed in epithelial and endothelial cells. ^##^*P* < 0.01 versus control; ^*∗∗*^*P* < 0.01 versus ALI.

To investigate the protective effect of Hox A9 on rat lung, the expression of HoxA9 was observed by using immunohistochemistry and real-time PCR. As shown in Figure 3(a), the protein expression of HoxA9 in the ALI group was increased (*P* < 0.01) at 24 hours. And MSCs infusion promoted the further increase. Moreover, the increase of HoxA9 expression in the ALI group or ALI + MSC group was mainly distributed in epithelial and endothelial cells. The results of real-time PCR also showed an obvious increase of Hox A9 mRNA expression in ALI or ALI + MSC group (Figure 3(b)), indicating that MSCs protected the cells from injures and Hox A9 promoted this protective effect of MSCs.

### 3.4. HoxA9 Promoted Proliferation of MSCs

**Figure 4:**
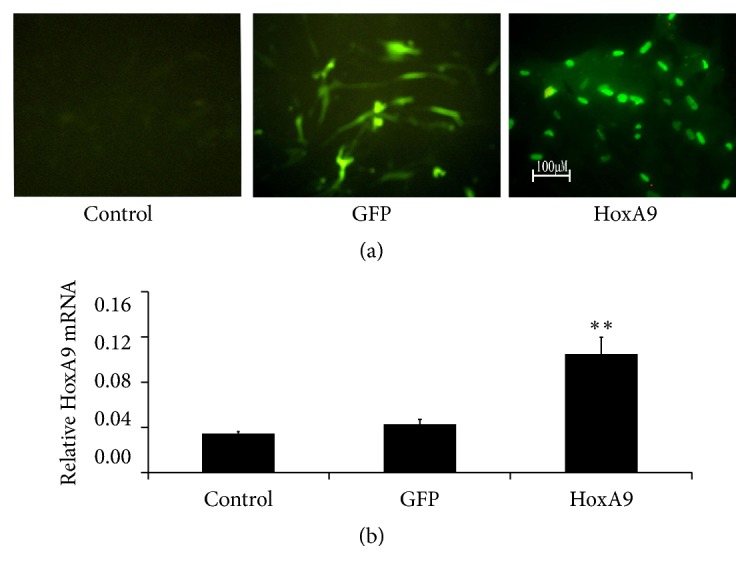
HoxA9 expression in lung was determined by fluorescence microscopy (a) and real-time PCR (*n* = 4, (b)). HoxA9 expression located in the nucleus and increased markedly at 24 hours after pLenO-Hox A9 infection. ^*∗∗*^*P* < 0.01 versus control or GFP.

To examine the effect of MSCs with or without Hoxa9 on wound repair, pLenO-HoxA9 lentiviral vector was constructed and transfected into MSCs. After 24 h transfection, the successful transfection was observed by fluorescence microscopy. The results showed that no fluorescence was observed in control group while visible fluorescence was observed in empty plasmid group which was located throughout the cells and HoxA9 group which was located in the nucleus (Figure 4). The results of real-time PCR also showed that HoxA9 was increased dramatically in Hox A9 group (*P* < 0.01) (Figure 4).

**Figure 5:**
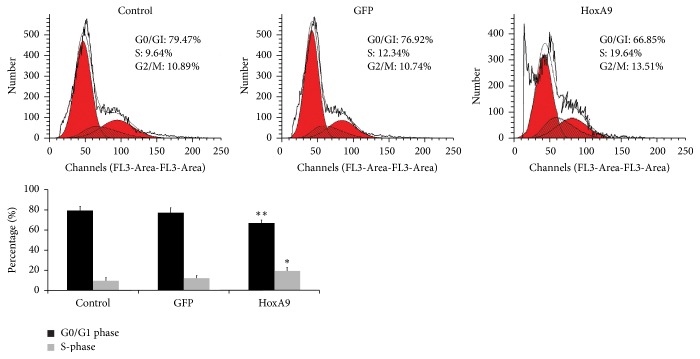
Proliferation of MSCs was determined by flow cytometry (*n* = 4). The results showed that the ratio of G0/G1 deceased, while the ratio of S phase cells was significantly increased in the pLenO-HoxA9 group. ^*∗*^*P* < 0.05, ^*∗∗*^*P* < 0.01 versus control or GFP.

Next, we observed the proliferation of MSCs by using flow cytometry. After transfection with pLenO-GFP or pLenO-HoxA9, the results of cell cycle showed that the ratio of G0/G1 deceased, while the ratio of S phase cells was significantly increased in the pLenO-HoxA9 group, indicating that Hox A9 overexpression can promote G0/G1 phase cells into S phase and MSC proliferation (Figure 5).

### 3.5. HoxA9 Inhibited Adhesion of MSCs with Leukocytes

**Figure 6:**
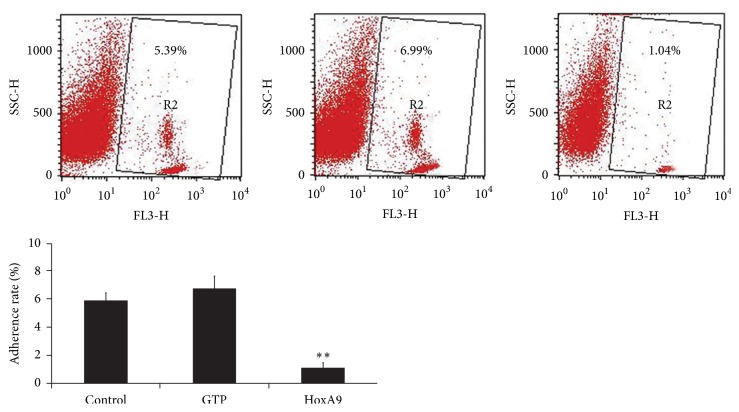
Adhesion of MSCs with leukocytes was determined by flow cytometry (*n* = 4). After infection with Hox A9, leukocyte adherence significantly decreased. ^*∗∗*^*P* < 0.01 versus control or GFP.

The adherence of leukocytes to MSCs showed no significant difference between control and empty vector group. However, after infection with pLenO-Hox A9, leukocyte adherence significantly decreased 6.7-fold (*P* < 0.01 compared to control) (Figure 6).

## 4. Discussion

ALI is a common critical disease in ICU. During ALI/ARDS, cell necrosis and apoptosis destroy the tight junctions between the cells and alveolar capillary blood membrane, inducing increased permeability [12]. Several studies showed that exogenous MSCs transplanted into body can differentiate into lung main cells such as alveolar epithelial cells and pulmonary vascular endothelial cells and play the role in repairing the damaged tissues. Van Haaften et al. [13] report suggests that MSCs can repair alveolar epithelial cells, promote lung surfactant secretion, and treat neonatal ARDS, indicating that MSCs in the lungs after implantation can differentiate to replace damaged alveolar epithelial cells and reduce the lung injury. Our in vivo study showed that MSCs had a protective effect on lung injury including increasing survival rate and reducing the inflammatory cells infiltration induced by LPS, reflecting an improvement in lung inflammation. Moreover, MSCs were widely reported to have an anti-inflammatory action in numerous studies [14, 15], suggesting that MSCs played a positive role in maintaining the integrity of lung cells.

Hox genes are an evolutionary highly conserved gene family, which determine the developmental fate of cells. To examine the expression Hox genes in ALI after MSC administration, real-time PCR array technology was carried out after 24 h of MSCs administration. The results showed that many Hox genes were differentially expressed. Among them, HoxA9 increased in ALI lungs at 24 hours after MSC administration more than tenfold. HoxA9 is a critical survival factor for both the endothelial and epithelial cells in the lung. Studies have shown that HoxA9 accelerated restoration of endothelial damage [16]. Except for the protective effects of HoxA9 on the endothelium, Hox A9 has also been reported to promote E-cadherin expression [17]. Furthermore, HOXA9 potently attenuated the expression of matrix metallopeptidase 9 (MMP 9) by controlling the binding of nuclear factor-kappa B to the promoter of MMP 9 genes, respectively [17]. It has been suggested that HoxA9 within the lung may therefore play a critical role in contributing to the regulation of alveolar-capillary permeability and promoting lung repair. In our study, we observed that MSC treatment increased Hox A9 expression in ALI lungs, which were distributed mainly in larger bronchial and blood vessels, and some were distributed in the distal lung epithelial cells, indicating that the distribution of Hox A9 is not limited to MSCs. Recently, more and more studies have shown that MSCs has a strong paracrine capacity and proposed it as the principal reason that contributes to the overexpression of Hox A9 in the lungs [18].

In this study, we constructed the Hox A9-overexpressed MSCs using lentiviral vector infection and then detected the distribution and Hox A9 mRNA expression in the cells. The results showed that HoxA9 was mainly located in the nucleus and the expression of HoxA9 mRNA after lentivirus transduction was dramatically increased, suggesting that lentivirus mediated HoxA9 expression in the MSCs was efficient and stable. Moreover, we also assessed the proliferation and adherent ability of the MSC-Hox A9. The results from the flow cytometry assay showed that the proliferation was increased and the inflammatory adherent abilities were decreased significantly compared with the normal MSCs.

## 5. Conclusion 

In conclusion, Hox A9-expressing character is required for MSCs to exert a therapeutic effect on ALI/ARDS. The underlying mechanism for the requirement of HoxA9 expressing character of MSCs in the treatment of ALI/ARDS may be partly related to the promoted proliferation and inhibitory inflammatory adhesion.

## Figures and Tables

**Table 1:** HOX gene expression levels in lungs after MSCs administration.

Gene	Fold changes			
	MSC versus control	ALI versus control	ALI + MSC versus control	ALI + MSC versus ALI
Hhex	2.34^*∗∗*^	−1.14	3.17^*∗∗*^	3.60^##^
Hoxa1	−1.27	2.55^*∗∗*^	3.13^*∗∗*^	1.23
Hoxa3	1.77	−1.01	−2.86^*∗∗*^	−2.86^##^
Hoxa5	2.09^*∗∗*^	−2.44^*∗∗*^	1.97	4.81^##^
Hoxa9	1.97	1.88	11.16^*∗∗*^	5.94^##^
Hoxb5	3.16^*∗∗*^	2.07^*∗∗*^	4.85^*∗∗*^	2.34^##^
Hoxb7	3.17^*∗∗*^	1.96	3.55^*∗∗*^	1.81
Hox c9	2.11^*∗∗*^	1.63	2.38^*∗∗*^	1.46
Hoxd8	1.42	1.32	−3.01^*∗∗*^	−4.00^##^
Lhx2	2.03^*∗∗*^	1.38	3.97^*∗∗*^	2.88^##^
Meis1	−1.04	2.97^*∗∗*^	1.47	−2.02^##^

^*∗∗*^ *P* < 0.01 versus control; ^##^*P* < 0.01 versus ALI.
